# Urocortin Role in Ischemia Cardioprotection and the Adverse Cardiac Remodeling

**DOI:** 10.3390/ijms222212115

**Published:** 2021-11-09

**Authors:** Eva M. Calderón-Sánchez, Débora Falcón, Marta Martín-Bórnez, Antonio Ordoñez, Tarik Smani

**Affiliations:** 1 Group of Cardiovascular Pathophysiology, Institute of Biomedicine of Seville, University Hospital of Virgen del Rocío/University of Seville/CSIC, 41013 Seville, Spain; ecalderon-ibis@us.es (E.M.C.-S.); dfalcon-ibis@us.es (D.F.); mmartin55@us.es (M.M.-B.); antorfernan@us.es (A.O.); 2 Department of Medical Physiology and Biophysics, University of Seville, 41009 Seville, Spain

**Keywords:** urocortin, adverse cardiac remodeling, cardioprotection, ischemia and reperfusion, heart failure

## Abstract

Despite the considerable progress in strategies of myocardial protection, ischemic heart diseases (IHD) and consequent heart failure (HF) remain the main cause of mortality worldwide. Several procedures are used routinely to guarantee the prompt and successful reestablishment of blood flow to preserve the myocardial viability of infarcted hearts from ischemia injuries. However, ischemic heart reperfusion/revascularization triggers additional damages that occur when oxygen-rich blood re-enters the vulnerable myocardial tissue, which is a phenomenon known as ischemia and reperfusion (I/R) syndrome. Complications of I/R injuries provoke the adverse cardiac remodeling, involving inflammation, mishandling of Ca^2+^ homeostasis, apoptotic genes activation, cardiac myocytes loss, etc., which often progress toward HF. Therefore, there is an urgent need to develop new cardioprotective therapies for IHD and HF. Compelling evidence from animal studies and pilot clinical trials in HF patients suggest that urocortin (Ucn) isoforms, which are peptides associated with stress and belonging to the corticotropin releasing factor family, have promising potential to improve cardiovascular functions by targeting many signaling pathways at different molecular levels. This review highlights the current knowledge on the role of urocortin isoforms in cardioprotection, focusing on its acute and long-term effects.

## 1. Introduction

**Figure 1:**
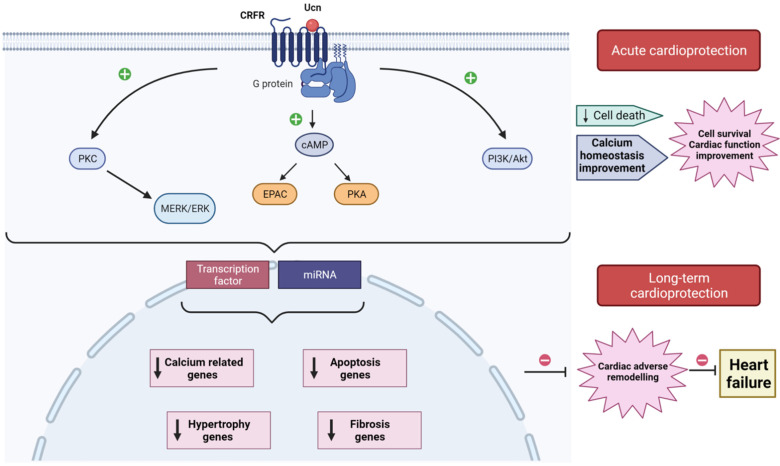
Schematic model illustrating acute and long-term cardioprotection afforded by urocortin (Ucn) from I/R injuries. In heart, Ucn binds to CRFR2, which interacts with G-proteins activating different signaling pathways (PKC-MERK/ERK; cAMP-EPAC/PKA; PI3K/Akt) that acutely decrease cell death, improve Ca^2+^ handling, enhance cell survival, and improve cardiac function. Ucn also activates transcription factors and stimulates miRNAs, release which regulates the expression of genes related to apoptosis, fibrosis, hypertrophy, and Ca^2+^ homeostasis. The downregulation of these genes prevents the development of adverse cardiac remodeling, avoiding its progress toward heart failure.

Despite the progress in cardiovascular disease treatment, the healthcare burden of ischemic heart failure (HF) is increasing worldwide [1]. Heart ischemia is originated by the critical obstruction of coronary arteries, leading to an imbalance between the consumption and supply of nutrients in the affected area in the heart. Ischemia causes cells lesions of different degrees depending on the extent of blood flow reduction and the length of the ischemic period, which influence the reduction in pH, ATP, and creatine phosphate, as well as increased levels of intracellular Na^+^ and Ca^2+^ concentrations, enlarged cell volume, and intracellular membranes disruption [2]. Treatment for myocardial ischemia involves the prompt and timely recovery of blood flow, which is known as myocardial reperfusion or revascularization and is necessary to save oxygen-deficient tissue (for a review, see [3,4]). Paradoxically, reperfusion causes additional injuries due to metabolites reaction with oxygen giving rise to reactive oxygen species (ROS) known as oxygen paradox [5]; stress of sarcoplasmic reticulum (SR), producing the accumulation of secondary metabolites and bad protein products; capillary non-reflow that leads to a worse local reperfusion [6,7,8]; and the mishandling of the intracellular Ca^2+^ concentration ([Ca^2+^]_i_) [9]. The pathophysiological value of [Ca^2+^]_i_ homeostasis is well-recognized. Actually, one of the critical factors involved in I/R syndrome is the cytoplasmic Ca^2+^ overload produced by the abnormal Ca^2+^ homeostasis. Isolated cardiac myocytes after I/R presents an increase in diastolic [Ca^2+^]_i_, a decrease in the systolic [Ca^2+^]_i_ transients, and a reduction of SR Ca^2+^ load, which correlate with a decrease in the activity of Na^+^/Ca^2+^ exchanger (NCX) [9,10]. All these events culminate in the mitochondrial permeability transition pore (MPTP) opening, apoptosis, and cell death [5] as illustrated in Figure 1. This phenomenon is widely known as I/R syndrome [11,12]. 

Sequelae of acute myocardial infarction (AMI) are known to trigger the adverse cardiac remodeling, which is the basic mechanism underlying the progression to HF considered the end stage of various types of cardiovascular disease, including ischemic heart disease (IHD) [13]. Therefore, there is an urgent need for adjunct cardioprotection therapy to prevent the impact of I/R injuries on heart function [14,15].

In the last two decades, Urocortin (Ucn) isoforms, peptides related to stress, arose as potential therapeutic drugs to improve performances of heart in I/R and under HF [16,17]. This review aims to highlight Ucn’s role in IHD and discusses the available clinical evidence of Ucn isoforms’ therapeutic feature in HF.

## 2. Structure and Expression of Urocortin

Ucn is a mammalian peptide member of the corticotropin-releasing factor (CRF) family. Three isoforms of Ucn have been described so far, Ucn1, Ucn2, and Ucn3 [18,19,20,21], which differ in terms of their structure, expression, and affinity to CRF receptors. Ucn1 is a 40 amino acid (aa) peptide having 63% and 45% sequences identity with urotensin and CRF, respectively [18]. Ucn1 was first identified in the brain, where it is expressed mainly in the Edinger–Westphal nucleus, but it was also located in the supraoptic nucleus, lateral olive, and lateral septum [18,22,23] and in other brain regions [24]. The expression of Ucn1 mRNA was also detected in the atrium and the ventricle of human heart. Similarly, the presence of Ucn1 peptide was demonstrated by immunocytochemistry assay in human heart [25]. Ucn1 was also detected at the mRNA and protein levels either in rat cardiac myocytes or fibroblasts [16,26] and in other peripheral tissues such as in the gastrointestinal system [27,28], the immune system, thymus, liver, adrenal gland, placenta, skin, and skeletal muscles [29,30]. 

Ucn2 is formed by 38 aa showing homologies with rat and human CRF (34%) and Ucn1 (43%). Meanwhile, Ucn3 (38–41 aa) has 37–40% homology with Ucn2 [19,20,21]. The expression of Ucn2 and Ucn3 was first studied using PCR that revealed their high expression in colon, small intestine, muscle, stomach, thyroid, adrenal, pancreas, spleen, and heart [20]. Particularly, experimental models using rodents showed that Ucn2 is also expressed in the central nervous system and peripheral tissues, being highly detected in cardiac myocytes [31], in skeletal muscle and skin [32]. Mouse Ucn3 mRNA expression was found in areas of the brain, small intestine, and skin. Different studies also demonstrated that neurons from the hypothalamus and amygdala also express Ucn3 [24]. Using RT-PCR and immunostaining, it was demonstrated that Ucn3 is also expressed in human heart [25,33,34]. 

## 3. Corticotropin Releasing Factor Receptors and Signaling Pathways

Ucn isoforms have high affinity to CRF receptors CRFR1 and CRFR2, which are G-protein coupled receptors that trigger various downstream signal transduction pathways. CRFR1 is mainly expressed in the brain, while CRFR2 is highly expressed in cardiac cells and peripheral tissue [35]. CRFR2 has three variants (α, β, γ), which vary in their N-terminal extracellular domains conferring differences in their subcellular localization [36]. CRFR2α was detected in all chambers of human heart, and CRFR2β was specifically identified in the left atrium [25,37]. Ucn isoforms and receptors are expressed within the left ventricle of the human myocardium at mRNA level, including CRFR1 and CRF [38]. Of note, this study showed a downregulation of CRFR2α and upregulation of CRFR1, CRF, and Ucn3 in diseased hearts excised from patients undergoing heart transplantation. In addition, a new splicing variant of CRFR1 gene named CRFR1j apparently is more expressed in HF patients than in healthy donor [38]. 

Structurally, CRFR1 and CRFR2 are approximately 70% identical at the aa level, but they exhibit considerable divergence at the N-terminal extracellular domain. Hence, they display ligand selectivity between Ucn members [24]. Indeed, Ucn1 binds with similar affinity to CRFR1 and CRFR2; meanwhile, Ucn2 and Ucn3 are selective ligands for CRFR2 [19,21]. CRFR2 interacts with Gs, Gq, and Gi proteins, activating different signaling pathways [39]. Independent studies demonstrated that the stimulation of CRFR2 contributes to the activation of cAMP and protein kinase A (PKA) signaling cascade [26], exchange protein activated by cAMP (Epac), extracellular signal-regulated kinase (ERK), and protein kinase C (PKC) [40]. For instance, experiments using ex vivo Langerdorff-perfused hearts and isolated cardiac myocytes showed that Ucn through CRFR2 induced potent positive inotropic and lusitropic effects, involving Epac, PKC. and mitogen-activated protein kinases (MAPK) signaling pathways [41]. CRFR2 stimulation also induced AMP-activated protein kinase (AMPK), phosphatidylinositol 3-kinase (PI3-K), and Akt kinase activation in isolated heart muscle and in vivo in intact hearts [31,42]. By contrast, CRFR2 antagonist markedly decreased the induced phosphorylation of PKA, CREB, CaMKII, and AKT [35].

Altogether, signaling pathways activated downstream CRFR2 play a critical role in different aspects of myocardial function, which explain the many effects that Ucn isoforms have on the cardiovascular system.

## 4. Acute Action of Urocortin in the Cardioprotection

The three isoforms of Ucn have established protective actions against myocardial I/R since they attenuate I/R incidences and the consequent cardiac adverse remodeling. The early studies about Ucn highlighted its acute effects in Langendorff-perfused mice, rat, and sheep hearts or using isolated neonatal rat ventricular myocytes (NRVM) and adult cardiac myocytes (for a review, see [43]). The acute administration of Ucn before ischemia or at the onset of reperfusion improved cardiac hemodynamic parameters, decreased the infarct size, and attenuated apoptosis assessed by TUNEL staining, caspase activity, or lactate dehydrogenase (LDH) [44,45,46,47]. Moreover, Ucn addition before ischemia and during reperfusion, but not only during reperfusion, significantly restored ATP and creatine phosphate in heart tissue, which are high-energy molecules required to prevent cell death after injury. Accordantly, Ucn inhibited creatine phosphokinase in similar conditions [45]. Similarly, the addition of Ucn before reperfusion prevented I/R-induced diastolic Ca^2+^ overload and recovered completely the amplitude of [Ca^2+^]_i_ transients; thus, it improved [Ca^2+^]_i_ handling and improved heart contractility [9]. Interestingly, cardiac cell incubation with Ucn2 inhibited I/R-upregulation of proteins related to the store-operated Ca^2+^ signaling pathway, such as Orai1, TRPC5, and STIM1 [48] (Figure 1). 

Interestingly, in addition to Ucn isoforms’ acute effects, increasing evidence demonstrated that they also have durable cardioprotective effects, attenuating events related with the adverse cardiac remodeling, such as the regulation of apoptotic genes and fibrosis. For instance, Ucn inhibited I/R-induced cardiac autophagy by Beclin1 downregulation through the PI3K/Akt pathway [49]. Ucn1 increased the expression of apoptotic genes CD40lg, Xiap, and BAD at mRNA and protein levels, in cells undergoing I/R through Epac2 and ERK1/2 activation [50]. Actually, this study demonstrated that Ucn1 promotes apoptosis programmed cell death to the detriment of necrosis, which will limit the impact of cardiac cell loss and posterior inflammatory processes in I/R [50]. In contrast, another study associated Ucn2 cardioprotective actions with the overexpression of the anti-apoptotic gene *Bcl-2* and the downregulation of pro-apoptotic genes *Bax* and *Bim* [51]. It is not yet clear in which situation Ucn isoforms activate or inhibit apoptotic genes under I/R, which is worth deeply investigating. In addition to apoptotic genes, Ucn also stimulated the upregulation of other genes, such as cardiotrophine-1 (CT-1) [46], potassium channel Kir 6.1 [52], glucocorticoid-responsive kinase-1 (SGK1) [53], or Orai1 and TRPC5 channels [48], suggesting that the cardioprotective action of Ucn isoforms involved a wide range of signaling pathways.

More recently, two independent studies showed that Ucn1-induced cardioprotection involved microRNAs (miRNAs) dysregulation in Langendorff-perfused heart subjected to global ischemia and reperfusion [54,55]. miRNAs are small non-coding RNAs that regulate a plethora of cellular processes related to the adverse cardiac remodeling, including cardiac myocyte apoptosis, necrosis, and fibrosis [56]. In fact, under I/R, Ucn1 upregulated miR-125a-3p, miR-324-3p, and downregulated miR-139-3p. Further experiments using NRVM demonstrated that the effect of Ucn1 involved the activation of CRFR2, Epac2, and ERK1/2. Furthermore, the overexpression of miR-125a-3p, miR-324-3p, and miR-139-3p modulated the expression of different genes involved in cell death and apoptosis (*BRCA1*, *BIM*, *STAT2*), in cAMP and Ca^2+^ signaling (*PDE4a*, *CASQ1*), in cell stress (*NFAT5*, *XBP1*, *MAP3K12*), and in cell metabolism (*CPT2*, *FoxO1*, *MTRF1*, *TAZ*). Similarly, Ucn2 was suggested to upregulate miRNA-221 in perfused heart, which inhibited apoptotic (*BIM*, *BMF*, *Ddit4*, *p27*) and autophagic genes (*LC3-II*) both at mRNA and protein levels [54].

Altogether, these data demonstrated a novel role of Ucn in myocardial protection, involving post-transcriptional regulation of genes through miRNAs [55].

## 5. Urocortin Role in the Adverse Cardiac Remodeling

The early adverse cardiac remodeling after an ischemic event is characterized by the alteration in [Ca^2+^]_i_ homeostasis, inflammation, and changes in the expression of different genes related to cell death, hypertrophy, and extracellular matrix components, particularly the generation of collagen, increasing fibrosis in the heart. These events evoke heart dysfunction, leading to HF [57].

In vivo studies have been performed to investigate the effects of Ucn administration in the adverse cardiac remodeling and cardiac function (Table 1). Most studies focused on the role of Ucn2 because it is highly specific for CRFR2, as mentioned before. Ucn2 administration (415 µg/kg/d) during 30 days, in mice post-AMI produced by permanent ligation of the left coronary artery, significantly decreased the infarct size, prevented the development of cardiac hypertrophy, reduced cardiac mass, and significantly decreased the expression of collagen 1 gene and fibrosis [58]. In another preclinical cardiac arrest model, the intravenous (i.v.) administration of Ucn2 (10 μg/kg) upon the onset of resuscitation ameliorated left ventricular systolic and diastolic functions and cardiac output, while it decreased cardiomyocyte apoptosis assessed by TUNEL assay. Ucn2 produced a significant increase in Akt, ERK, and STAT3 activation and phosphorylation in the myocardium after cardiac arrest and resuscitation [59]. The activation of Akt is considered protective, since it is known to sequester the pro-apoptotic protein BAD in the cytosol, reducing the levels of free BAX and inhibiting the activation of apoptosis [60]. 

In a rat model of I/R, the i.v. infusion of Ucn2 (150 µg/Kg) right before heart reperfusion recovered significantly cardiac contractility and prevented fibrosis. Ucn2 administration recovered left ventricle ejection fraction and shortening, improved the amplitude of [Ca^2+^]_i_ transient, and modulated the expression of several proteins related to [Ca^2+^]_i_ homeostasis, such as TRPC5 and Orai1 channels [48]. Moreover, Ucn2 infusion in the same animal model prevented I/R dysregulation of miR-324-3p and miR-139-3p expression 1 week after I/R, which regulates the expression of different genes related to adverse remodeling. These studies confirmed that Ucn2 might provide long-lasting effects through post-transcriptional genes’ modulation via miRNAs [55]. 

Interestingly, a recent study demonstrated that the Ucn3 gene transfected using adeno-associated viruse 8 (AAV8) in mice after 3 weeks of AMI improved Ca^2+^ handling and left ventricle function, as compared with HF mice. In addition, mRNA expression of hypertrophy markers and stress as BNP, ANF, α-skeletal actin, and β-MHC was lower in mice transfected with Ucn3 gene than in HF mice, preventing adverse cardiac remodeling and apoptosis [61].

## 6. Therapeutic Values of Urocortin in Heart Failure

Preclinical studies proposed a positive therapeutic potential of Ucn’s family in cardiovascular diseases since they increased cardiac output, left ventricular ejection fraction (LVEF) [62,63], and evoked vasodilation in human coronary arteries isolated from ischemic HF patients [64]. Therefore, the first investigations studied Ucn’s value as a biochemical marker for the diagnosis and management of patients with HF [65,66,67] but also as therapeutic drug that might overcome HF symptoms [68], as summarized in Table 2. Clinical and translational studies about Ucn in AMI patients are still rare, although several reports investigated Ucn in HF patients, as reviewed recently [68]. One of the first clinical trials in HF patients with reduced ejection fraction (HFrEF) identified elevated levels of Ucn1, as compared to non-HF patients. The study demonstrated significant positive relationships between plasma Ucn1 levels and other circulating neurohormones known to be activated in this condition, such as ANP, NT-proANP, BNP, NT-proBNP, C-type natriuretic peptide, adrenomedullin, and endothelin 1 [67]. Plasma levels of Ucn1 in AMI patients are apparently higher than in control patients during the first 5 days after the heart attack. The high plasma level of Ucn1 on day 0 confirmed a significant and independent predictive value for mortality and adverse cardiac events. Of note, plasma Ucn1 levels are significantly elevated both in patients with non-ST elevation AMI (NSTEMI) and with ST elevation AMI (STEMI), comparing to control [69,70].

In the case of Ucn2, increased blood levels were observed in patients with systolic dysfunction but not in those with coronary artery disease without myocardial infarction [71]. A recent report used a new immunoassay for plasma NT-proUcn2 and observed modest but significant increased plasma concentrations in patients with HFrEF from different origins, including those with myocardial infarction, along with an inverse relationship to 2-year mortality in HF. This study concluded that perhaps the modest increase in NT-proUcn2 concentrations in HF may limit its utility as a diagnostic marker, but further investigations are required to confirm this statement [66].

On the other hand, independent reports analyzed the effect of the i.v. infusion of Ucn on hemodynamic parameters in healthy and HF patients. For instance, that i.v. administration of a bolus of 50 µg of Ucn1 to healthy volunteers, compared to placebo administration, resulted in increased plasma levels of corticotropin (ACTH), cortisol, ANP, and decreased in ghrelin level [72]. By contrast, Ucn1 infusion did not alter plasma levels of second messengers as cAMP, BNP, adrenaline, noradrenaline, endothelin, plasma renin activity, aldosterone, etc. [72]. Moreover, the administration of Ucn1 in patients with stable congestive heart failure (CHF) also increased levels of corticotropin and cortisol as compared to the placebo group, but without differences in ANP and ghrelin levels. Nevertheless, Ucn1 infusion did not change the heart rate, systolic and diastolic blood pressure, or the cardiac output [62,72]. No detectable differences in hemodynamic and renal effects were detected, either [62].

Other studies evaluated the use of i.v. administration of Ucn2 to avoid Ucn1 effects on the central nervous system. For example, the i.v. administration of Ucn2 (25 and 100 µg) in healthy volunteers and in patients with congestive HF increased cardiac output, heart rate, and LVEF in a dose-dependent manner; meanwhile, it decreased systemic vascular resistances [63]. Similar findings on hemodynamic responses were observed in patients with congestive HF in NYHA II-III receiving the same doses of Ucn2 [73]. Later, a randomized controlled clinical trial using Ucn2 in acute decompensated HF, confirmed systemic vasodilation, and increased cardiac output without increasing the heart rate. Ucn2 administration was accompanied by a sustained fall in BNP levels and a transient decline in renal indices with a reduction in creatinine clearance, which was probably due to the pronounced falls in systemic arterial pressure [74]. Another clinical trial evaluated the effect of Ucn2 and Ucn3 in healthy volunteers and in patients with stable HF who did not respond to concomitant medical therapy. The study found that Ucn2 and Ucn3 (360 pg/min and 12 ng/min, respectively) caused vasodilatation and reduced peripheral vascular resistances; meanwhile, they increased the heart rate and cardiac output [75]. The author stated that Ucn3 exerted more marked hemodynamic effects than Ucn2 in HF patients but not in healthy subjects. However, the heart rate increase with the highest dose of Ucn3 was less pronounced in patients with HF, which was probably due to concomitant β-adrenoreceptor blocker therapy according to the authors’ conclusion [75]. No episodes of arrhythmia or adverse effects, such as those previously witnessed in animal models, were observed in this study [75]. Interestingly, the i.v. infusion of Ucn3 (15 and 30 ng/kg/min) in HFrEF patients induced a significant increase in cardiac index following 60 min infusion and reduced systemic vascular resistances without significant effects on heart rate or systolic blood pressure [76].

## 7. Conclusions

To summarize, even if the effect of Ucn infusion in patients suffering AMI has not been addressed yet, data from clinical trials in HF patients provided evidence that Ucn isoforms efficiently decrease peripheral vascular resistances and the mean arterial pressure. The lowering of peripheral vascular resistances will be beneficial for AMI patients since it may promote a decrease in the afterload. At the same time, cardiac output increase by Ucn might be beneficial to compensate for cardiac myocyte loss during AMI, although this effect may also increase the oxygen consumption of the myocardium. Moreover, Ucn’s ability to regulate the post-transcriptional and translational processes involved in cardiac remodeling is worth being deeply investigated. Actually, understanding the mechanisms and downstream targets of Ucn in preclinical studies holds promise to allow fine-tuning of the signaling pathway activated under I/R, which can be hopefully translated soon to clinical trials. Future detailed clinical trials using a large number of patients are eagerly needed to determine whether Ucn could be useful as a sensitive biomarker for the adverse cardiac remodeling post-AMI or as therapeutic drugs that will mitigate AMI injuries to avoid the evolution of adverse cardiac remodeling toward HF.

## Figures and Tables

**Table 1:** Preclinical studies using different protocol to administrate Ucn2 in ischemic models. * gc/Kg indicates genome copies of adenovirus administrated/Kg.

Animal Model	Dosage	End Points	Reference
Mice post-AMI	415 μg/Kg/d *Ucn2* (30 days)	▪ Infarct size decrease ▪ Mean arterial pressure decrease ▪ Cardiac hypertrophy prevention ▪ Cardiac mass reduction ▪ Markers of cardiac remodeling decrease	[58]
Preclinical cardiac arrest rat model	10 μg/Kg i.v. *Ucn2*	▪ Acute hemodynamic instability improvement ▪ Infarct size decrease ▪ Left ventricular systolic and diastolic functions improvement ▪ Cardiac output improvement ▪ Cardiomyocyte apoptosis decrease	[59]
Rat model of I/R	150 µg/Kg i.v. *Ucn2*	▪ Left ventricle ejection fraction and shortening recovery ▪ Cardiac contractility recovery ▪ Fibrosis prevention ▪ Infarct size decrease	[48]
Rat model of I/R	50, 150, or 300 µg/Kg *Ucn2*	▪ Left ventricle ejection fraction and shortening recovery	[55]
Mice post-AMI	1.9 × 10^13^ gc/Kg * AAV8.*UCn3*	▪ Cardiac contractility and relaxation improvement ▪ Decrease in markers of cardiac remodeling	[61]

**Table 2:** Completed clinical trials that used Ucn isoforms.

Clinical Study Design and State	Patients	Dosage	Outcomes	Negative Outcomes	Reference
Ucn1 vs. placebo in a balanced, randomized, single-blind, cross-over design	8 healthy unmedicated men	Ucn1, 50 μg (1 μg/mL)	▪ Normotension ▪ Plasma ghrelin suppression ▪ cAMP, BNP, Nt-pro-BNP, adrenaline, noradrenaline, ADM, GH, LH, FSH, PRL, AVP, ET-1, PRA, or aldosterone plasma levels unaltered	Temporal ACTH and cortisol increase	[72]
Ucn1 vs. placebo in a balanced, randomized, single-blind, cross-over design	8 males with stable congestive HF (LVEF ≥40%), NYHA class II–III and creatine <0.15 mM. With medication	Ucn1, 50 μg (1 μg/mL)	▪ cAMP, BNP, Nt-pro-BNP, adrenaline, ghrelin, noradrenaline, ADM, GH, LH, FSH, PRL, AVP, ET-1, PRA or aldosterone plasma levels unaltered ▪ LVDV and LVSV, LVEF and mitral valve Doppler indices unaltered ▪ Unaltered urine volume and urinary excretion of Ucn1, Na^+^, K^+^, CrCl, and cAMP	Temporal ACTH and cortisol increase	[62]
Single-blind dose-escalation design	8 males with stable congestive HF (LVEF ≥40%), NYHA class II–III. With medication	Ucn2, 25–100 μg (0.5–2 μg/mL)	▪ cAMP plasma levels increase ▪ CO and HR increase ▪ MAP and VR decrease ▪ LVEF increase at high dose (100 μg) ▪ Electrocardiogram alteration and arrhythmias not observed	Temporal flushed during drug infusion	[63]
Single-blind dose-escalation design	8 healthy unmedicated men	Ucn2, 25–100 μg (0.5–2 μg/mL)	▪ cAMP and cGMP plasma levels increase ▪ CO and HR increase dose dependent ▪ DBP and MAP and VR decrease dose dependent ▪ LVEF increase dose dependent ▪ PRA, Ang II, norepinephrine, cGMP increase and epinephrine decrease at high dose ▪ ACTH, cortisol, insulin, ghrelin, Nt pro-BNP, arginine vasopressin, ET-1, or ADM unaltered	Urine Na^+^, K^+^, and CrCl decrease	[73]
Single-center, randomized, double-blind, placebo-controlled trial	53 HF patients (LVEF <40%)	Ucn2, 400 μg (2 μg/mL)	▪ CO increase ▪ SBP, DBP and VR decrease ▪ Plasma Na^+^ and K^+^ unaffected ▪ Plasma levels of ANP and BNP decrease	Temporal flushed during infusion Hypotension Urine volume and CrCl reduced	[74]
Randomized study	8 healthy patients and 8 HF patients (LVEF <35%), NYHA functional class II–III	Ucn2, (3.6–36 pmol/min) Ucn3, (360–3600 pmol/min)	▪ Vasodilation ▪ VR decrease and CO increase ▪ SBP and DBP in HF patients decrease and DBP in healthy decrease with Ucn3	Tachycardia at Ucn3 high dose	[75]
Randomized, double-blind, placebo-controlled, parallel-group, ascending dose study	15 healthy patients and 45 HF patients (LVEF ≤35%), NYHA functional class II–IV	Human stresscopin (5, 15, 30 ng/Kg/min)	▪ CI increase ▪ DBP and VR decrease	Erythema and hot feeling	[76]

Abbreviations: ACTH, adrenocorticotropic hormone; ADM, adrenomedullin; ANP, atrial natriuretic peptide; AngII, angiotensin II; AVP, arginine vasopressin BNP, brain natriuretic peptide; cAMP, cyclic adenosine monophosphate; cGMP, cyclic guanine monophosphate; CO, cardiac output; CI, cardiac index; CrCl, creatinine clearance; DBP, diastolic blood pressure; ET-1, endothelin-1; FSH, follicle-stimulating hormone; GH, growth hormone; HR, heart rate; LVEF, left ventricular ejection fraction; LH, luteinizing hormone; LVDV, left ventricular diastolic volume; LVSV, left ventricular systolic volume; MAP, mean arterial pressure; NYHA, New York Heart Association; Nt-pro-BNP, N-terminal pro-brain natriuretic peptide PRA, plasma renin activity; PRL, prolactin; SBP, systolic blood pressure; TSH, thyroid stimulating hormone; VR, vascular resistance.

## Data Availability

Not applicable.

## Abbreviations

AC	adenylate cyclase
ACTH	corticotropin
ANP	atrial natriuretic peptide
AMI	acute myocardial infarct
AMPK	AMP-activated protein kinase
AAV	adeno-associated viruses
cAMP	cyclic AMP
BNP	brain natriuretic peptide
CHF	congestive heart failure
CRF	corticotropin-releasing factor
CRF-R1,2	corticotropin-releasing factor-receptor 1,2
CT-1	cardiotrophin 1
Epac	exchange protein directly activated by cAMP
ERK1/2	extracellular signal–regulated kinases 1/2
HF	heart failure
HFrEF	reduced ejection fraction
IHD	ischemic heart diseases
I/R	ischemia/reperfusion
LDH	lactate dehydrogenase
LVEF	left ventricular eyection fraction
LVDP	left ventricular developed pressure
LVEDP	left ventricular end diastolic pressure
MAPK	mitogen activated protein kinase
miRNAs	microRNAs
MPTP	mitochondrial permeability transition pore
NRVM	neonatal rat ventricular myocytes
NCX	Na^+^/Ca^2+^ exchanger
PI3-K	phosphatidylinositol 3-kinase
PKA	protein kinase A
PKC	protein kinase C
ROS	reactive oxygen species
SGK1	glucocorticoid-responsive kinase-1
SR	sarcoplasmic reticulum
STEMI	myocardial infarction with ST segment elevation
Ucn1, 2, 3	urocortin 1, 2, 3
